# Diversity and origins of bacterial and archaeal viruses on sinking particles reaching the abyssal ocean

**DOI:** 10.1038/s41396-022-01202-1

**Published:** 2022-03-02

**Authors:** Elaine Luo, Andy O. Leu, John M. Eppley, David M. Karl, Edward F. DeLong

**Affiliations:** 1 Daniel K. Inouye Center for Microbial Oceanography: Research and Education (C-MORE), University of Hawaiʻi at Manoa, Honolulu, HI 96822 USA; 2 Present Address: Woods Hole Oceanographic Institution, 266 Woods Hole Road, MS 51, Woods Hole MA, 02543 Falmouth, USA; 3 Present Address: Australia Center for Ecogenomics, University of Queensland, St. Lucia QLD, 4072 Australia

**Keywords:** Metagenomics, Biogeochemistry, Microbial biooceanography, Microbial ecology, Microbial biooceanography

## Abstract

Sinking particles and particle-associated microbes influence global biogeochemistry through particulate matter export from the surface to the deep ocean. Despite ongoing studies of particle-associated microbes, viruses in these habitats remain largely unexplored. Whether, where, and which viruses might contribute to particle production and export remain open to investigation. In this study, we analyzed 857 virus population genomes associated with sinking particles collected over three years in sediment traps moored at 4000 m in the North Pacific Subtropical Gyre. Particle-associated viruses here were linked to cellular hosts through matches to bacterial and archaeal metagenome-assembled genome (MAG)-encoded prophages or CRISPR spacers, identifying novel viruses infecting presumptive deep-sea bacteria such as *Colwellia*, *Moritella*, and *Shewanella*. We also identified lytic viruses whose abundances correlated with particulate carbon flux and/or were exported from the photic to abyssal ocean, including cyanophages. Our data are consistent with some of the predicted outcomes of the viral shuttle hypothesis, and further suggest that viral lysis of both autotrophic and heterotrophic prokaryotes may play a role in carbon export. Our analyses revealed the diversity and origins of prevalent viruses found on deep-sea sinking particles and identified prospective viral groups for future investigation into processes that govern particle export in the open ocean.

## Introduction

Microbial processes are fundamental to productivity and export in the oceans. Microbes fuel the biological carbon pump by reducing inorganic carbon to organic carbon, some of which sinks into the deep sea in aggregates of both organic and inorganic particulate matter [1, 2]. These sinking particles and their associated microbes play a critical role in the global carbon cycle by sequestering approximately 4 gigatons of our planet’s atmospheric carbon annually [3], roughly equivalent to the total standing carbon stock in marine biomass [4]. Sinking particles represent “hotspots” of microbial activity, harboring diverse assemblages that play active roles in the transformation of organic matter in the oceans [5–11]. These microbial communities connect the surface and deep oceans [12] and fuel biogeochemical cycling through selective remineralization of labile organic carbon [13].

Previous studies of microbial assemblages associated with sinking particles have focused predominantly on cellular organisms (e.g., bacteria, archaea, and eukaryotes). Bacteria and archaea account for a majority of cellular rRNA reads observed on sinking particles that reach the open ocean’s abyss [10, 14]. Due to enriched yet variable nutrient composition, as well as heterogeneous microscale habitat space, particle-attached prokaryotic assemblages can be more active and diverse than planktonic assemblages [15–20]. Sinking particles are enriched in larger “copiotrophic” bacteria typically associated with gut microbiomes, such as Bacteroidetes, δ-, ɛ-, and γ-proteobacterial groups [8–11, 15–20].

While cellular assemblages on particles are becoming more well-studied [9, 10, 15–22], there still are relatively few studies of viral diversity on sinking particles. Although microscopic analyses indicate that viruses can be abundant on sinking particles, their identity and role in carbon flux have remained largely undetermined [23, 24]. Exploring their genetic diversity could identify novel viral populations associated with sinking particles. Furthermore, identifying whether and which particle-attached viruses might be exported from shallower waters relates to two proposed conceptual models that invoke opposite effects on particle export. The “viral shunt” model proposes that viral lysis attenuates carbon export processes, versus the “viral shuttle” model that proposes virus enhancement of export (reviewed in [25, 26]). In the viral shunt model, viruses enhance the microbial loop by transforming living cells into dissolved and particulate organic matter, which increases the availability of substrates for heterotrophic respiration in the upper ocean [27–29]. In the viral shuttle model, viruses enhance export from the surface to the deep ocean by lysing cells, releasing sticky material, such as polymers, proteins, and DNA [30], and promoting aggregation, leading to larger particles for more efficient export [25]. It is likely that a combination of both models applies to how viruses influence the marine environment, possibly depending on host metabolism, size, habitat, and viral reproductive strategies.

Identifying viruses that correlate with export flux has potential to identify key viral groups and mechanistic processes that influence carbon export. Validating mechanistic frameworks attempting to explain how viruses influence marine ecosystems, however, requires both laboratory experiments and field studies. Laboratory studies to date have focused on viruses of cultured eukaryotic hosts, which provided evidence that specific viruses can cause host death, aggregation, and sinking of large eukaryotic phytoplankton [23, 24, 31–33]. Smaller picoplankton have been thought to contribute little to particle export due to their small size and slow sinking velocities [34, 35], but more recently their contributions were starting to be recognized [36, 37]. These reports motivate exploration of the viral shunt/shuttle hypotheses in the open ocean, where picoplankton dominate primary production [38]. In the field, two studies have reported relationships between modeled carbon flux and relative abundances of viruses with predicted hosts of both phytoplankton [39] and picoplankton primary producers [40], while two others reported direct virus-driven aggregation of phytoplankton exported into the mesopelagic ocean using direct carbon flux measurements [41, 42].

Identifying viruses on sinking particles, inferring their origins and host associations, and determining which viruses correlate with particle export, can inform our understanding of microbial processes that influence carbon export in the ocean. Here, we report on the diversity and presumptive origins of viruses found on sinking particles at Station ALOHA in the North Pacific Subtropical Gyre (NPSG), an environment characteristic of oligotrophic open oceans that cover roughly 40% of our planet [43]. Deep-moored sediment traps have been deployed at Station ALOHA since 1992 [44], providing rich time-series data on particulate export flux. Three years of sediment trap samples collected from 2014 to 2016 at 4000 m were analyzed to study viruses associated with sinking particles exported to abyssal depths with implications for carbon burial and sequestration. Metagenomic data from sediment trap samples [10, 14] were used to assemble 857 deep trap viruses (DTVs) that represent some of the most prevalent particle-associated viruses sinking to 4000 m in the NPSG. To overcome challenges in identifying novel viruses without sequence representation in current databases, viral populations were linked to their cellular hosts using bacterial and archaeal metagenome-assembled genomes (MAGs) previously determined from the same samples [14]. Viral diversity and virus-host dynamics were explored over a 3 year time-series [10, 14], revealing the depth of origins of vertically transported viruses, and specific viral populations that correlated with carbon export flux. The DTV data and analyses reported here provide new perspectives on the diversity and origins of viruses on sinking particles in the open ocean and implications for carbon export.

## Methods

A schematic overview of our workflow is presented in Fig. S1.

### Sample collection, extraction, and sequencing

Station ALOHA (22°45’ N, 158° W) is a relatively seasonally stable environment located in the NPSG and is the sampling site of the Hawaii Ocean Time-series (HOT) program [45]. Metagenomes previously generated from sinking particles collected in a deep-moored sediment trap at 4000 m [10, 14, 44] were used here to assemble DTVs. A total of 63 samples spanning 3 years from 2014 to 16 were used to generate metagenomic and particulate carbon flux data [10, 14]. Sediment trap set-up, deployment, recovery, and sample processing for measuring particulate carbon flux have been previously described [10, 14, 44]. For reference and consistent with previous studies, samples that displayed ≥ 150% of the 28-year mean carbon flux were considered summer export pulse samples [14, 44]. Extraction, sequencing, read quality-control (QC), and assembly of sediment trap metagenomes were previously described [10, 14]. Read sequence data are available on NCBI SRA under Bioproject PRJNA482655.

### Virus-specific reassembly

Viral contigs and associated reads were identified using four methods: (i) ≥ 3 kb contigs were filtered using VIRSorter v1.03 [46] using the virome database, and 11,610 viral contigs from all categories were retained (Table S1). (ii) ≥ 1 kb contigs were filtered using VIBRANT [47], and 15,356 viral contigs were retained (Table S1). (iii) 43,663 contigs from 121 metagenome-assembled genomes (MAGs) from the same samples [14] were filtered through VIRSorter and VIBRANT, and 1123 viral contigs identified from either program were retained. (iv) 1470 Eukaryotic viruses were collected from NCBI and de-replicated using cd-hit-est at ≥ 95% across ≥ 90%. BWA-MEM v0.7.15 (Li 2013) and msamtools [48] was used to identify reads mapping to putative viral contigs at ≥ 95% identity across ≥ 45 bp or to dereplicated NCBI eukaryotic viruses at ≥ 70% identity across ≥ 45 bp. 24 million total viral reads were reassembled using metaSPAdes v3.13.1 [49], which was chosen due to improved genome recovery and low rate of generating false apparent circularity [50].

### DTV database curation

Viral contigs were first identified and retained if they met one or more of the following three criteria: (i) The contig was classified as viral by both VIRSorter and VIBRANT (767 contigs). (ii) The contig was classified as viral by either VIRSorter or VIBRANT, and contained a phage marker protein (≥ 30 bit score to capsid, head, neck, tail, spike, portal, terminase, clamp loader, T4 proteins, T7 proteins, Mu proteins, excisionase, phage integrase, repressor protein CI, or Cro). 537 contigs were identified as viral by VIRSorter and contained one or more phage marker proteins. 1396 contigs were identified as viral by VIBRANT and contained one or more phage marker proteins. If a contig contained prophages identified from VIRSorter and VIBRANT that differed in length, the shorter prophage sequence was retained prior to de-replication. (iii) If the contig contained one or more eukaryotic virus marker protein, at ≥30 bit score to protein domains from NCLDV capsids, envelope, and Poxvirus proteins (674 contigs). Proteins were predicted using Prodigal v2.6.3 [51] and functionally annotated using HMMer v.3.2 [52] against the PFAM-A v30 database [53].

Subsequently, all 3374 viral contigs identified from any of the above methods were clustered with cd-hit-est v4.6 [54] at ≥ 95% ANI across ≥ 50% resulting in 2359 non-redundant viral population genomes. To focus on full genomes or large genomic fragments, only ≥ 10 kbp contigs were retained for the final 857 populations that form the DTV database. PFAM functional annotations were inspected to ensure that no ribosomal proteins were present, with the exception of S21, which was previously found in a cultivated *Pelagibacter* phage [55]. The high proportion of novel viral diversity in our samples precludes using reference genomes for the detection of chimeras, which are expected to occur at a frequency of ~0.5% using metaSPAdes [50]. Sequences were inspected for chimeras through self-alignment using LAST v1021 [56] to identify repeats at ≥ 95% ANI across ≥ 5 kbp. 3 populations displayed this signature and were noted as chimeras (Table S2). DTV sequences are available under NCBI PRJNA482665.

### Genomic completion

109 complete, non-redundant virus genomes (Table S2) were identified by looking for terminal repeats indicating apparent circularity [57]: 83 were identified using Virsorter, 4 from check_circularity.pl [58], and 32 using NUCmer v3.1 [59] to find direct terminal repeats 20–5000 bp in length within 200 bp of both ends [60].

### Identifying DTVs

Host taxonomy was assigned to 95 DTVs using these four methods, ordered by confidence and priority (Table S2): (i) 57 DTVs were identified through alignments to putative prophages in MAGs with known taxonomic annotations (Table S3). An initial set of 105 DTVs aligned to MAG scaffolds at ≥ 95% identity across ≥ 50% of the viral contig to account for possible circular permutations. A final 57 DTV-MAG links were retained after independently confirming that the MAG bin’s assigned taxonomy, based on multiple scaffolds, was consistent with taxonomic annotations of the individual MAG scaffold that aligned to the virus. MAG scaffolds were annotated using LAST against the Genome Taxonomy DataBase (GTDB) release 04-RS89 [61], and the scaffold was considered consistent with the bin’s annotation if the scaffold recruited the greatest number of protein hits from the bin’s assigned genus or family. Inconsistencies between MAG scaffold and bin annotations may be due to several potential, and non-mutually exclusive, reasons including: mis-annotation of the virus due to sparse representation in existing databases; mis-binned viral contigs in the MAG bins; and viruses with previously unrecognized broad host ranges. (ii) 14 DTVs were linked to cellular hosts using CRISPR spacers identified from MAGs with known taxonomic annotations. CRISPR repeats and spacers were identified using CRASS [62] from reads mapping MAGs at ≥ 97% identity across ≥ 75% of the read length. Viruses were linked to MAGs if the CRISPR repeat sequence matched the MAG at 100% nucleotide identity and a blastn e-value of ≤ 1e-10, and if the entire CRISPR spacer matched the viral sequence at 100% nucleotide identity. (iii) A total of 37 and 1 DTVs were identified based on protein alignments to GTDB and to RefSeq96, respectively [63]. Predicted virus proteins were aligned using LAST and taxonomy was broadly assigned if viruses contained ≥ 50% proteins with hits at ≥ 60% amino acid identity (AAI) to a single genus-level group. (iv) 3 DTVs were identified based on nucleic acid alignments (≥ 95% identity across ≥ 50%) to annotated viruses in the ALOHA2.0 viral database ([64], Table S4).

To identify the number of novel viral populations recovered from our samples, DTV proteins were aligned using LAST to RefSeq96 [63], and in the following marine viral metagenomic databases available as of 2020: uvMED [65], uvDEEP [66], GOV [67], EV [68], MED2017 [69], ALOHA 2.0 [64], Nishimura 2017 [57], Coutinho 2017 [70], and GOV2.0 [71]. For a conservative estimate on the number of novel populations, populations were considered novel if they did not meet broad taxonomic assignments at ≥60% AAI across ≥50% of proteins to any reference genome or contig (Table S2). Annotated viral proteins were considered novel if they contained PFAM domains not found in previously reported datasets [64, 67, 72] (Table S5).

A total of 184 temperate phages were identified using four methods. (i) Functional annotations identified 94 populations with temperate phage markers (≥ 30 bit score to integrase, excisionase, Cro, or CI repressor). (ii) 46 populations shared significant homology (≥ 95% identity across at least of half the viral contig) with prophages identified by VIRSorter and VIBRANT from original assemblies. (iii) VIRSorter and VIBRANT respectively identified 49 and 110 final viral populations as prophages. (iv) 48 viruses linked to MAGs shared ≥ 95% ANI to a contiguous MAG chromosomal region and the aligning MAG scaffold was ≥10 kb longer than the virus, consistent with cellular regions expected to flank integrated prophages. Viral populations displaying none of these four characteristics were inferred to be lytic.

### Recovering cellular metagenome-assembled genomes (MAGs) used in viral host identification

The MAGs used here were previously assembled, quality controlled, and analyzed as previously described [14]. Assembled MAGs are available under NCBI SAMN14675689-SAMN14675809.

### Temporal abundance and persistence

Reads from each sample were mapped using BWA-MEM and filtered using msamtools at ≥ 95% identity across ≥45 bp for viruses, and ≥ 97% identity across ≥ 45 bp for MAGs. Anvi’o v3 [73] was used to calculate coverage profiles for every sample, using interquartile range (IQR) coverage, which diminishes the effect of conserved or hypervariable regions in respectively over- and under-estimating coverage. IQR coverage normalized to the smallest library size (normalized coverage) was used to approximate relative abundances (Tables S6, S7). A population is considered present in a sample if it displayed a non-zero IQR coverage (Table S2).

### Vertical transport and depth of origin

Metagenomic reads from sediment trap samples collected at 150 m in 2015 (NCBI PRJNA358725, [21]) and 2017 (NCBI PRJNA596510), and virioplankton samples collected from 5 to 500 m in 2014–16 (NCBI PRJNA352737, [64]), were mapped to DTVs at ≥ 95% identity across ≥ 80% of the read. Populations with non-zero IQR coverage in reads from 5 to 500 m were inferred to have been exported from the photic zone (Table S2). To determine viral depth of origin, 5–500 m virioplankton reads were mapped using BWA-MEM and filtered using msamtools at ≥ 95% identity across ≥ 80%. Populations were assigned based on the highest normalized IQR coverage to the surface (5–75 m), DCM (100–125 m), transitional (150–250 m) or mesopelagic (500 m) depth of origin. Populations with zero IQR coverage with reads recruited from this 5 to 500 m dataset were classified as bathypelagic (Table S2).

### Viral correlation with particulate carbon export flux

The WGCNA package in R [74] was used to identify 194 viral populations belonging to one of three modules that displayed significant Pearson’s correlation (*p* < 0.05, [75]) with log-transformed particulate carbon flux (Table S2). Viruses were grouped into modules using the dynamic hybrid method with tree cut height at 0.988.

## Results and Discussion

**Fig. 1:**
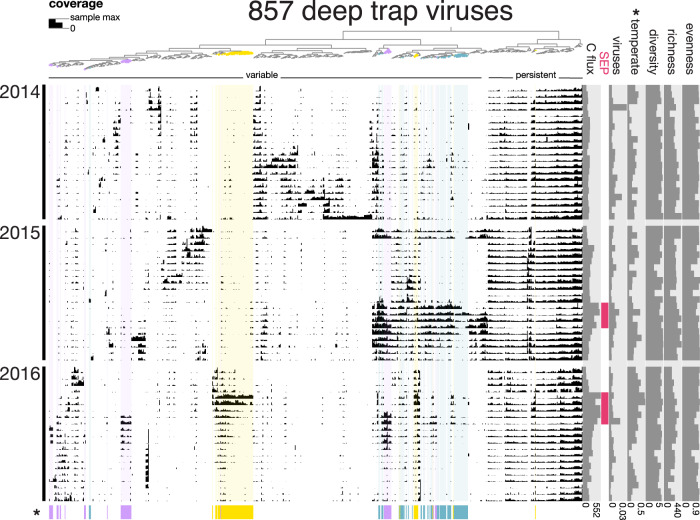
Coverage abundance profiles of 857 DTV populations across 63 samples. Each node on the top dendrogram and its associated column represents the coverages of one viral population. The top dendrogram clusters populations based on similarity in coverage abundance profiles. The top row indicates groups that appear to be variable or persistent across samples. Each row represents an individual sample, ordered by time. The height of black bars represents log IQR coverage for each population, normalized to the maximum in that sample. The right panels display sample biogeochemical data: particulate carbon export flux (µmol/m^2^/day), summer export pulse samples, proportion of total reads mapping to DTVs, abundance-normalized proportion of temperate DTVs, and Shannon’s diversity, richness, and evenness. Three WGCNA clusters of viruses positively correlated with carbon export flux are highlighted in color on the top dendrogram, on the background, and on bottom bars. Asterisks indicate variables that significantly correlate with carbon flux: proportion of temperate phages and WGCNA clusters.

Metagenomic data previously generated from sixty-three 4000 m sediment trap samples (collected at Station ALOHA between 2014 and 2016 [10, 14]) were used to identify viruses associated with deep-sea sinking particles. Sediment trap metagenome assembly, classification, virus-specific reassembly and curation recovered 857 viral populations forming the DTV database (Fig. S1). A total of 184 DTVs were tentatively identified as temperate phage, using genomic markers for lysogeny (total relative abundances shown on right panel of Fig. 1). Since only 0.8% of DTVs appeared derived from eukaryotic viruses (7 total), we focused our analyses on prokaryote viruses, 95 of which were linked to hosts through MAGs and reference databases. Previously reported particulate carbon export flux data [14, 44] indicated that these samples spanned the summer export pulse events in 2015 and 2016 (Fig. S2, described in [14]).

Particle-associated viruses from Station ALOHA were largely novel with respect to other marine environments, as 735 (86%) of DTVs were distinct from previously sequenced viruses (Table S2, [57, 63–71]). Of the remaining 122 DTVs similar to previously reported phage at ≥ 60% amino acid identity (AAI) across ≥ 50% proteins, 23 populations were similar to those previously observed from 5 to 500 m at Station ALOHA [64]. A total of 112 populations were similar to marine viruses collected from other environments, suggesting wider distribution of a few conserved viral groups. Only one population was similar at the genus level to the taxonomically annotated RefSeq96 database (a vibriophage), further highlighting the extent of novel genetic diversity observed in DTVs.

The DTVs contained 115 protein functional domains not previously found in virus datasets [64, 67, 72], including predicted protein domains involved in carbohydrate metabolism (Table S4). This predicted functional diversity in auxiliary metabolic genes might reflect metabolic pathways of cellular hosts on sinking particles, and viral strategies that supplement host carbon metabolism during infection []. For example, five DTVs (two identified as temperate) with unknown host associations contained predicted proteins involved in cellulose, pectate, and trehalose degradation, potentially reflecting the metabolism of copiotrophic hosts previously observed on sinking particles [14]. One DTV containing two predicted cellulose biosynthesis proteins was identified as temperate and linked to *Halomonas*. Another DTV containing a predicted chitin synthase was identified as a eukaryotic virus using protein markers (below). Only 7 DTVs contained predicted protein domains characteristic of eukaryotic viruses (NCLDV capsids, envelope, chitin, josephin, Poxvirus, and Baculovirus proteins), likely reflecting both their lower cell densities and larger, more complex and less readily assembled genomes. Taxonomic identification for these putative eukaryotic viruses was largely sparse or inconsistent. One DTV consistently shared homology with a virus of a crustacean (42/108 proteins to *Penaeus*), while two DTVs shared homology with capsids from phytoplankton viruses (chlorovirus at 26% AAI and *Feldmannia* virus at 31% AAI).

### Host taxonomic diversity of viruses on sinking particles reaching the abyssal ocean

**Fig. 2:**
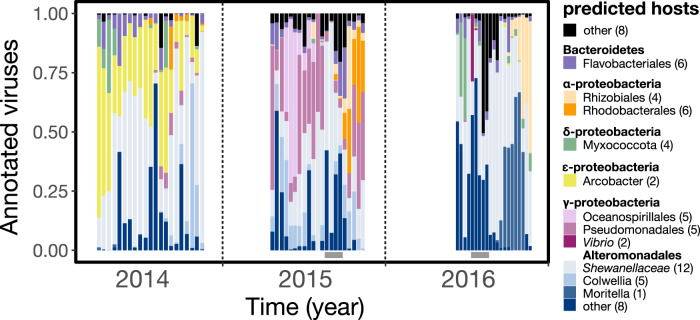
Relative abundances through time of 68 host-associated deep trap viruses (DTVs), identified by alignments to putative prophages and CRISPRs in MAGs. Relative abundances were calculated using IQR coverage normalized to a total of 1. Viral host annotations are grouped by color and host clade, with the number of viral populations in each group in parentheses. Bottom grey bars indicate summer export pulse samples based on particulate carbon export flux.

By aligning viral sequences to putative prophages or CRISPR spacers in MAGs reconstructed from the same samples [14], 68 DTVs were linked to host genome signatures. These linkages revealed broad taxonomic diversity in viruses and their hosts associated with sinking particles, including representation from Bacteroidetes, α-, δ-, ɛ-, and γ-proteobacterial groups (Fig. 2). In general, viruses infecting hosts in the Alteromonadales group (γ-proteobacteria) dominated sinking particles, frequently accounting for over half of the relative abundances of all annotated viruses. Of the Alteromonadales phages, DTVs linked to close relatives of deep-sea bacteria (e.g., *Shewanella, Colwellia*, and *Moritella*; [76]) were particularly abundant, mirroring the previously reported prevalence of these bacteria on sinking particles and in the deep sea [10, 14, 77]. In particular, one *Moritella* phage accounted for over half of all annotated DTVs observed in late 2016, consistent with anomalously high relative abundance of *Moritella* during this time period [14]. Other abundant viruses include two phages of *Arcobacteraceae* (ɛ-proteobacteria) that accounted for over half of all annotated DTVs observed in early 2014. These results are consistent with previous observations of abundant heterotrophs found on sinking particles in this environment [8, 10] and in other environments following cyanobacterial blooms [78].

### Viral correlation with particulate carbon export flux

**Fig. 3:**
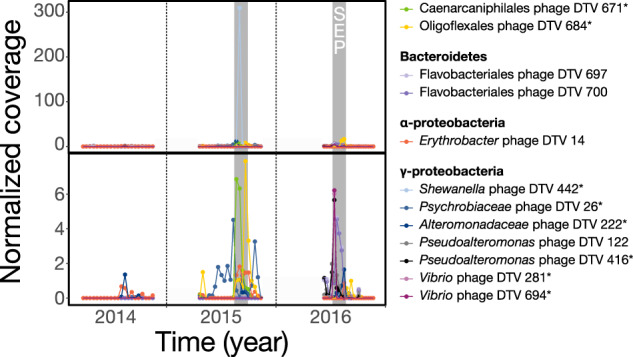
Normalized abundances of annotated viruses that significantly correlated with carbon flux. Of the 194 viral populations belonging to WGCNA clusters that significantly correlated with particulate carbon export flux, 12 were annotated using alignment to MAGs (high-confidence, denoted by asterisks) or the GTDB protein database. The bottom panel is a subset of the top panel, with the abundant *Shewanella* phage DTV 442 removed. Abundances were approximated using IQR coverage normalized to the smallest sequenced sample. Grey shading indicates summer export pulse samples based on particulate carbon export flux.

A total of 194 DTVs belonged to one of three WGCNA clusters that significantly correlated with particulate carbon export flux (Pearson *p* < 0.05, Fig. 1, highlighted groups). Twelve viral populations were annotated using alignment to prophages or CRISPR spacers in MAGs or to the GTDB protein database, with abundant representation from γ-proteobacterial groups (Fig. 3). In particular, one *Shewanella* phage was an order of magnitude more abundant than other viruses at the start of the 2015 summer export pulse. Other viral groups that correlated with carbon flux include phages infecting Caenarcaniphilales, Oligoflexales, Flavobacteriales, Psychrobiaceae, *Pseudoalteromonas*, and *Vibrio*. A total of 7 of the 8 MAG-linked DTVs had predicted hosts that either positively correlated with carbon flux and/or were enriched in the summer export pulse [14]. These carbon-flux-correlating hosts included members of the *Alteromonadaceae*, Caenarcaniphilales, Oligoflexales, *Pseudoalteromonas*, *Psychrobiaceae*, and *Vibrio*. These results are also consistent with a 2015 report of the presence of *Pseudoalteromonas* and *Vibrio* species correlating with carbon flux estimates from optical data [40]. Of 194 DTVs that correlated with particulate carbon flux, 131 lacked temperate phage markers and were presumably lytic, which is consistent with a predicted outcome of the viral shuttle hypothesis. Our results provide a new insight into virus-mediated export processes: viral lysis of heterotrophic hosts, which remineralize organic matter on sinking particles, could enhance export efficiency.

### Evidence of vertical transport of viruses on sinking particles

**Fig. 4:**
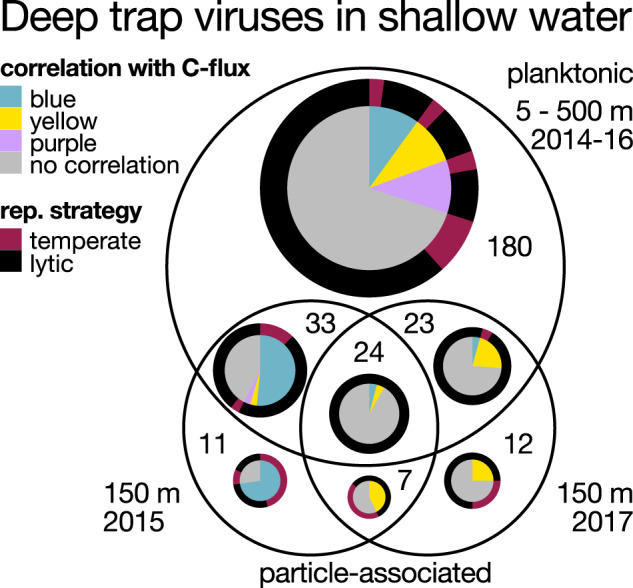
Characteristics of 290 DTVs observed in shallower water. Totals represent the number of DTVs that recruited reads (non-zero interquartile coverage) from either one, or combinations of, three shallow-water planktonic (free-living) or sinking particle-associated samples. The inner pie chart indicates membership in one of three groups (blue, yellow, purple) that correlated with particulate carbon export flux, as identified by WGNCA (Table S2). The outer ring indicates viral reproductive strategy, as identified by the presence of prophage markers (PFAM bit score > = 30, Table S2). The pie chart area scales linearly to the number of DTVs in each category.

A total of 290 DTV populations recruited reads (non-zero interquartile coverage) from previously characterized near-surface metagenomes: particle-associated samples collected at 150 m during 2015 and 2017 [21], and virioplankton samples collected from 5 to 500 m [64] during 2014–2016 (Fig. 4). Previous observations of oligotrophic virioplankton at Station ALOHA (not associated with sinking particles) indicated that these planktonic viral populations were generally specific to surface, DCM, and mesopelagic depths, similar to their hosts. Little evidence existed for putative eurybathic viruses inhabiting depth ranges throughout the water column [64, 72]. Accordingly, the 290 planktonic viral populations observed in ≤ 500 m time-series samples that were also found in the sinking particle-associated sediment trap samples at 4000 m were presumably transported to the abyss via sinking particles. Some of these DTVs correlated with carbon flux, particularly in populations that were observed in shallow-water particle-associated samples (Fig. 4). DTVs having predicted temperate or lytic reproductive strategies were both observed on shallow-water sinking particles (Fig. 4). Our results are consistent with a predicted outcome of the viral shuttle hypothesis, in particular, the observation of surface-water derived lytic viruses on sinking particles reaching deep sea during periods of high carbon flux.

Taxonomic diversity of the predicted hosts of vertically-transported DTVs were consistent with known shallow-water bacteria previously observed on the same particle-associated deep trap samples, such as Gammaproteobacteria, Verrucomicrobia, and Cyanobacteria [21], the latter group one of the most abundant primary producers at Station ALOHA [79]. Furthermore, five presumably vertically-transported DTVs were linked to MAG hosts that were enriched during periods of high carbon flux, such as Caenarcaniphilales, Gammaproteobacteria, and Oligoflexales [14].

**Fig. 5:**
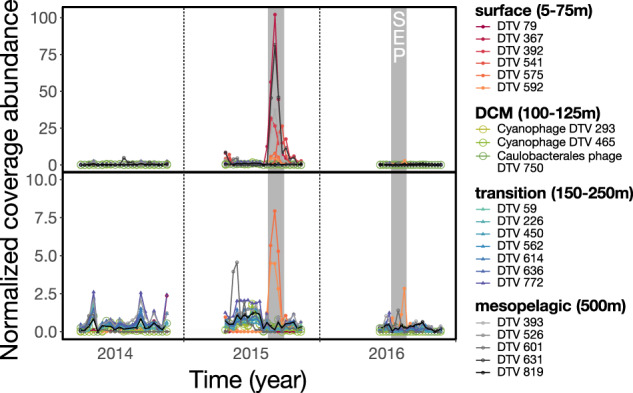
Predicted abundances from 2014 to 2016 of 21 DTVs with inferred depths of origins in the upper 500 m. The top panel includes all mapping data. The bottom panel represents a subset of the top panel, with the most abundant viruses removed. Abundances were approximated using IQR coverage normalized to the smallest sequenced sample. Grey shading indicates summer export pulse samples based on particulate carbon export flux. Surface (5–75 m), deep chlorophyll maximum (100–125 m), transition zone (150–250 m), and mesopelagic (500 m).

Alignments between DTVs and viral contigs assembled from 5 to 500 m virioplankton samples [64] revealed 21 DTVs that occurred in shallower waters during the same period (Table S4). Most of these 21 populations were observed only at specific depths in the upper 500 m, indicating that they originated in the upper ocean (Fig. S3). The summed normalized abundances of these viruses (Fig. 5) also significantly correlated with carbon flux (Pearson’s correlation *p* = 0.01), consistent with vertical transport of these viral populations on sinking particles. Additionally, all three annotated viruses were identified as lytic and associated with bacteria in the upper water column, such as autotrophic cyanobacteria and heterotrophic Caulobacterales (Fig. 5 DCM, S3, Table S2), the latter of which was enriched in SEP samples [14] and associated with phytoplankton blooms [78]. Overall, our results are consistent with a previous report of correlations between cyanophages and carbon export [40] and suggest that lytic viruses of picoplankton might play a role in particle export in the open ocean. Our observations provide strong evidence that viruses were transported from the photic zone to the deep sea on sinking particles. Future investigation of the rate and mechanism(s) underlying viral contribution to particle export, perhaps coupled with in situ incubations to target specific viral groups, will help constrain viral effects on global biogeochemical cycles.

### Depth of origin of viruses on sinking particles

**Fig. 6:**
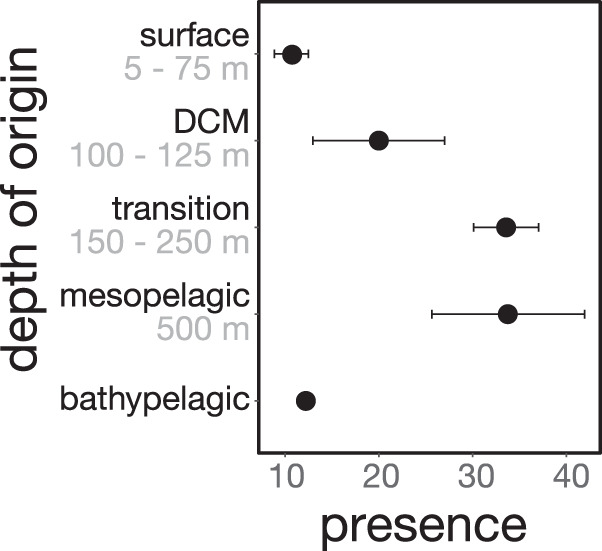
Presence of pelagic viral populations in 63 deep trap samples grouped by the populations’ inferred depths of origin. A population was considered present in a sample if it recruited reads across > 25% of its sequence (> 0 IQR coverage). Surface (5–75 m), deep chlorophyll maximum, 100–125 m), transition zone (150–250 m), mesopelagic (500 m), and bathypelagic (4000 m).

Of the 21 DTVs that aligned to virioplankton population genomes from the upper ocean, 6, 3, 7, and 5 DTVs were respectively assigned to surface (5–75 m), DCM (deep chlorophyll maximum, 100–125 m), transitional (150–250 m) or mesopelagic (500 m) depth of origin (Fig. 5, Table S2). Viruses that originated from ≥ 150 to 500 m were more frequently found in exported particles relative to those from above 150 m (Fig. 6). This may in part reflect the higher turnover rates that occur in upper waters as predicted by the Martin curve [80, 81]. Taken together, viruses throughout the upper 500 m were observed on sinking particles exported to the deep ocean at 4000 m, and viruses that originated below the DCM were associated most frequently with particle export. Although the bulk of the SEP has been attributed to large primary producers such as diazotrophic diatom associations originating from the near-surface ocean [14, 44], we observed a frequent but low background signal of viruses originating below the mixed layer depth in sediment trap samples. Our results indicate microbial colonization and subsequent viral infection and host lysis as particles descended through the mesopelagic.

### Viral infection patterns in bathypelagic bacteria

Virus-host links revealed variable abundance patterns between DTV and MAG populations. Some virus-host populations displayed tightly coupled abundance co-variance, such as a putative *Shewanella* phage and its host (Fig. S4a, left). Such similarity suggests that these viruses may have infected and integrated into the genomes of nearly all of the host population, and remained stable as a prophage in host populations captured in sinking particles over three years. Similar read coverages in both viral and cellular regions in the aligning *Shewanella* scaffold (Fig. S4a, right) further reflect roughly equal predicted abundance between the integrated prophage and the host genome. The DTV could represent a rarely induced prophage, or possibly a degraded prophage element no longer capable of induction or excision.

In contrast, some virus-host pairs displayed decoupled abundance profiles. For example, a *Moritella* phage population was nearly absent in 2014–2015, but then mirrored host abundances in late 2016 (Fig. 2b, left). Its absence in 2014 is evident by sparse to no coverage in the aligning viral region in the *Moritella* scaffold, a large discrepancy compared with the ~20x coverage shown in non-viral scaffold regions (Fig S4b, right). As another example, one *Arcobacter* phage that was abundant in sinking particles in 2014 was undetectable in 2015 and 2016 (Fig S4c, left). Its absence in 2015 is evident in the near-zero coverage in aligning viral regions in the MAG scaffold, a large discrepancy compared to the ~50x coverage observed in non-viral MAG scaffold regions (Fig. S4c, right). Interestingly, other shorter regions on this MAG scaffold seem to also have disappeared in 2015. Upon closer inspection, one of these regions contained an integrase and two contained transposases, both of which are common features of island regions that diversify rapidly relative to the rest of the genome (reviewed in [82]). These results likely reflect the heterogeneity and spatiotemporal patchiness of sampled host populations.

Sediment trap samples accumulate sinking particles from potentially heterogeneous sources over a 10–14 day period. Thus, variability between virus and host abundances does not necessarily reflect viral integration or excision within the same host population. Considering that particles in open-ocean environments sink at variable speeds [83], originate from diverse sources [10, 14], and can be carried by horizontal advection up to tens of kilometers per day [84], sediment trap samples likely reflect microbial communities from heterogeneous sources across variable temporal and spatial scales. Despite the source, time, depth, space-integrated nature of our dataset, we observed strong differential patterns in virus and host abundances. Such strong patterns reflect a high level of viral and host microdiversity and dynamic viral-host interactions in the open ocean, even within genetically similar populations.

We observed no evidence of prophage induction and replication, since no viral population was at any time point much more abundant than their host. Prophage induction and replication might not have been detected due to the following reasons. (i) Sinking particles are composed of heterogeneous organic and inorganic materials, including marine snow and fecal pellets (reviewed in [85]). (ii) Microhabitats on sinking particles might lead to spatially asynchronous prophage induction that is difficult to identify from the bulk nucleic-acid signatures sampled in sediment traps. Temporally asynchronous induction would be blurred by the time-integrated nature of our samples (10–14 days) and variable particle sinking rates [83]. (iii) If a portion of a prophage population is induced and replicating, differences in copy number could break assemblies between an integrated prophage and host genome, leading to an underrepresentation of inducible prophages in MAGs. Taken together, identifying virus-host links using alignments to putative prophages in MAGs might be biased towards inactive temperate phages found at similar abundances to that of their hosts.

## Conclusions

To study the diversity of sinking particle-associated viruses and their relationship to carbon export, we assembled and curated a database of 857 DTVs collected from sediment traps at 4000 m in an open ocean environment characteristic of the largest biome on Earth. To identify viruses that lack sequenced relatives in reference databases, cellular MAGs assembled from the same samples were used to link 68 DTVs to their hosts. Using these linkages, we identified novel viral populations infecting deep-sea bacteria, and found that viral diversity on sinking particles was highly variable across a three-year period. The relative abundances of some DTV populations displayed positive correlations with particulate carbon flux, including viruses infecting heterotrophic bacteria that remineralize organic matter on sinking particles. Our results introduce a new consideration to viral impacts on particle export, in that virus-induced heterotrophic host mortality might enhance export efficiency. Some lytic DTVs were transported on sinking particles from the photic zone, revealing viral groups that might influence particle export, such as cyanophages that infect abundant primary producers in the open ocean. Our observations are consistent with a predicted outcome of the viral shuttle hypothesis. We further hypothesize that key viruses infecting both autotrophic and heterotrophic prokaryotes might enhance the export of carbon from the surface to the abyssal ocean and represent candidates for future investigations into mechanistic evidence for virus-mediated processes impacting particle export in the ocean.

## Supplementary information

Supplementary figures+legends, supplementary table legends

Table S1

Table S2

Table S3

Table S4

Table S5

Table S6

Table S7

## References

[1] McCave IN (1975). Vertical flux of particles in the ocean. Deep-Sea Res.

[2] Ducklow HW, Steinberg DK, Buesseler KO (2001). Upper ocean carbon export and the biological pump. Oceanography.

[3] Siegenthaler U, Sarmiento JL (1993). Atmospheric carbon dioxide and the ocean. Nature.

[4] Bar-On YM, Phillips R, Milo R (2018). The biomass distribution on Earth. Proc Natl Acad Sci USA.

[5] Turley CM, Mackie PJ (1994). Biogeochemical significance of attached and free-living bacteria and the flux of particles in the NE Atlantic Ocean. Mar Ecol Prog Ser.

[6] Turley CM, Stutt ED (2000). Depth-related cell-specific bacterial leucine incorporation rates on particles and its biogeochemical significance in the Northwest Mediterranean. Limnol Oceanogr.

[7] Aristegui J, Gasol JM, Duarte CM, Herndl GJ (2009). Microbial oceanography of the dark ocean’s pelagic realm. Limnol Oceanogr.

[8] Fontanez KM, Eppley JM, Samo TJ, Karl DM, DeLong EF (2015). Microbial community structure and function on sinking particles in the North Pacific Subtropical Gyre. Front Microbiol.

[9] Pelve EA, Fontanez KM, DeLong EF (2017). Bacterial succession on sinking particles in the ocean’s interior. Front Microbiol.

[10] Boeuf D, Edwards BR, Eppley JM, Hu SK, Poff KE, Romano AE (2019). Biological composition and microbial dynamics of sinking particulate organic matter at abyssal depths in the oligotrophic open ocean. Proc Natl Acad Sci USA.

[11] Preston CM, Durkin CA, Yamahara KM (2020). DNA metabarcoding reveals organisms contributing to particulate matter flux to abyssal depths in the North East Pacific ocean. Deep-Sea Res Part II.

[12] Mestre M, Ruiz-González C, Logares R, Duarte CM, Gasol JM, Sala MM (2018). Sinking particles promote vertical connectivity in the ocean microbiome. Proc Natl Acad Sci USA.

[13] Jiao N, Herndl GJ, Hansell DA, Benner R, Kattner G, Wilhelm SW (2010). Microbial production of recalcitrant dissolved organic matter: Long-term carbon storage in the global ocean. Nat Rev Microbiol.

[14] Poff KE, Leu AO, Eppley JM, Karl DM, DeLong EF (2021). Microbial dynamics of elevated carbon flux in the open ocean’s abyss. Proc Natl Acad Sci USA.

[15] DeLong EF, Franks DG, Alldredge AL (1993). Phylogenetic diversity of aggregate‐attached vs. free‐living marine bacterial assemblages. Limnol Oceanogr.

[16] Rieck A, Herlemann DPR, Jürgens K, Grossart HP (2015). Particle-associated differ from free-living bacteria in surface waters of the Baltic Sea. Front Microbiol.

[17] Crespo BG, Pommier T, Fernández-Gómez B, Pedrós-Alió C (2013). Taxonomic composition of the particle-attached and free-living bacterial assemblages in the Northwest Mediterranean Sea analyzed by pyrosequencing of the 16S rRNA. Microbiologyopen.

[18] Eloe EA, Shulse CN, Fadrosh DW, Williamson SJ, Allen EE, Bartlett DH (2011). Compositional differences in particle-associated and free-living microbial assemblages from an extreme deep-ocean environment. Environ Microbiol Rep..

[19] Ghiglione JF, Mevel G, Pujo-Pay M, Mousseau L, Lebaron P, Goutx M (2007). Diel and seasonal variations in abundance, activity, and community structure of particle-attached and free-living bacteria in NW Mediterranean Sea. Micro Ecol.

[20] López-Pérez M, Kimes NE, Haro-Moreno JM, Rodriguez-Valera F (2016). Not all particles are equal: The selective enrichment of particle-associated bacteria from the Mediterranean Sea. Front Microbiol.

[21] Farnelid H, Turk-Kubo K, Ploug H, Ossolinski JE, Collins JR, Van Mooy BAS (2019). Diverse diazotrophs are present on sinking particles in the North Pacific Subtropical Gyre. ISME J.

[22] Mende DR, Boeuf D, DeLong EF (2019). Persistent core populations shape the microbiome throughout the water column in the North Pacific Subtropical Gyre. Front Microbiol.

[23] Proctor LM, Fuhrman JA (1991). Roles of viral infection in organic particle flux. Mar Ecol Prog Ser.

[24] Peduzzi P, Weinbauer MG (1993). Effect of concentrating the virus‐rich 2‐2nm size fraction of seawater on the formation of algal flocs (marine snow). Limnol Oceanogr.

[25] Weinbauer MG (2004). Ecology of prokaryotic viruses. FEMS Microbiol Rev.

[26] Zimmerman AE, Howard-Varona C, Needham DM, John SG, Worden AZ, Sullivan MB (2019). Metabolic and biogeochemical consequences of viral infection in aquatic ecosystems. Nat Rev Microbiol.

[27] Wilhelm SW, Suttle CA (1999). Viruses and nutrient cycles in the sea. Bioscience..

[28] Gobler CJ, Hutchins DA, Fisher NS, Cosper EM, Sañudo-Wilhelmy SA (1997). Release and bioavailability of C, N, P, Se, and Fe following viral lysis of a marine chrysophyte. Limnol Oceanogr.

[29] Middelboe M, Jørgensen NOG, Kroer N (1996). Effects of viruses on nutrient turnover and growth efficiency of noninfected marine bacterioplankton. Appl Environ Microbiol.

[30] Alldredge AL, Silver MW (1988). Characteristics, dynamics and significance of marine snow. Prog Oceanogr.

[31] Shibata A, Kogure K, Koike I, Ohwada K (1997). Formation of submicron colloidal particles from marine bacteria by viral infection. Mar Ecol Prog Ser.

[32] Yamada Y, Tomaru Y, Fukuda H, Nagata T (2018). Aggregate formation during the viral lysis of a marine diatom. Front Mar Sci.

[33] Lawrence JE, Suttle CA (2004). Effect of viral infection on sinking rates of Heterosigma akashiwo and its implications for bloom termination. Aquat Micro Ecol.

[34] Michaels A, Silver M (1988). Primary production, sinking fluxes and the microbial food web. Deep-Sea Res. Part I.

[35] Richardson TL (2019). Mechanisms and pathways of small-phytoplankton export from the surface ocean. Ann Rev Mar Sci.

[36] Richardson T, Jackson GA (2007). Small phytoplankton and carbon export from the surface ocean. Science.

[37] Lomas MW, Moran SB (2011). Evidence for aggregation and export of cyanobacteria and nano-eukaryotes from the Sargasso Sea euphotic zone. Biogeosciences.

[38] Liu H, Nolla HA, Campbell L (1997). Prochlorococcus growth rate and contribution to primary production in the equatorial and subtropical North Pacific Ocean. Aquat Micro Ecol.

[39] Kaneko H, Blanc-Mathieu R, Endo H, Chaffron S, Delmont TO, Gaia M, et al. Eukaryotic virus composition can predict the efficiency of carbon export in the global ocean. iScience. 2021;24:102002. 10.1016/j.isci.2020.102002 PMC7811142 33490910

[40] Guidi L, Chaffron S, Bittner L, Eveillard D, Larhlimi A, Roux S (2015). Plankton networks driving carbon export in the oligotrophic ocean. Nature.

[41] Laber CP, Hunter JE, Carvalho F, Collins JR, Hunter EJ, Schieler BM (2018). Coccolithovirus facilitation of carbon export in the North Atlantic. Nat Microbiol.

[42] Sheyn U, Rosenwasser S, Lehahn Y, Barak-Gavish N, Rotkopf R, Bidle KD (2018). Expression profiling of host and virus during a coccolithophore bloom provides insights into the role of viral infection in promoting carbon export. ISME J.

[43] Karl DM, Church MJ (2014). Microbial oceanography and the Hawaii Ocean Time-series programme. Nat Rev Microbiol.

[44] Karl DM, Church MJ, Dore JE, Letelier RM, Mahaffey C (2012). Predictable and efficient carbon sequestration in the North Pacific Ocean supported by symbiotic nitrogen fixation. Proc Natl Acad Sci USA.

[45] Karl DM, Lukas R (1996). The Hawaii Ocean Time-series (HOT) program: Background, rationale and field implementation. Deep-Sea Res Part II.

[46] Roux S, Enault F, Hurwitz BL, Sullivan MB (2015). VirSorter: mining viral signal from microbial genomic data. PeerJ..

[47] Kieft K, Zhou Z, Anantharaman K (2020). VIBRANT: automated recovery, annotation and curation of microbial viruses, and evaluation of viral community function from genomic sequences. Microbiome.

[48] Arumugam M, Harrington ED, Raes J, Foerstner KU, Arumugam M, Bork P (2010). SmashCommunity: A metagenomic annotation and analysis tool. Bioinformatics..

[49] Bankevich A, Nurk S, Antipov D, Gurevich AA, Dvorkin M, Kulikov AS (2012). SPAdes: a new genome assembly algorithm and its applications to single-cell sequencing. J Comput Biol.

[50] Roux S, Emerson JB, Eloe-Fadrosh EA, Sullivan MB (2017). Benchmarking viromics: An in silico evaluation of metagenome-enabled estimates of viral community composition and diversity. PeerJ..

[51] Hyatt D, Chen G, Locascio PF, Land ML, Larimer FW, Hauser LJ. Prodigal: Prokaryotic gene recognition and translation initiation site identification. BMC Bioinformatics. 2010;11:119. 10.1186/1471-2105-11-119 PMC2848648 20211023

[52] Eddy SR (2011). Accelerated Profile HMM Searches. PLoS Comput Biol.

[53] Finn RD, Tate J, Mistry J, Coggill PC, Sammut SJ, Hotz H, et al. The Pfam protein families database. Nucleic Acids Res. 2008;36:281–8. 10.1093/nar/gkm960 PMC2238907 18039703

[54] Li W, Godzik A (2006). Cd-hit: A fast program for clustering and comparing large sets of protein or nucleotide sequences. Bioinformatics..

[55] Mizuno CM, Guyomar C, Roux S, Lavigne R, Rodriguez-Valera F, Sullivan M (2019). Numerous cultivated and uncultivated viruses encode ribosomal proteins. Nat Commun.

[56] Kielbasa SM, Wan R, Sato K, Kiebasa SM, Horton P, Frith MC (2011). Adaptive seeds tame genomic sequence comparison. Genome Res.

[57] Nishimura Y, Watai H, Honda T, Mihara T, Omae K, Roux S (2017). Environmental viral genomes shed new light on virus-host interactions in the ocean. mSphere..

[58] Imai T sprai = single pass read accuracy improver [Internet]. 2013. Available from: http://zombie.cb.k.u-tokyo.ac.jp/sprai/

[59] Kurtz S, Phillippy A, Delcher AL, Smoot M, Shumway M, Antonescu C (2004). Versatile and open software for comparing large genomes. Genome Biol.

[60] Beaulaurier J, Luo E, Eppley JM, Uyl PDen, Dai X, Burger A (2020). Assembly-free single-molecule sequencing recovers complete virus genomes from natural microbial communities. Genome Res.

[61] Parks DH, Chuvochina M, Waite DW, Rinke C, Skarshewski A, Chaumeil PA (2018). A standardized bacterial taxonomy based on genome phylogeny substantially revises the tree of life. Nat Biotechnol.

[62] Skennerton CT, Imelfort M, Tyson GW (2013). Crass: Identification and reconstruction of CRISPR from unassembled metagenomic data. Nucleic Acids Res.

[63] O’Leary NA, Wright MW, Brister JR, Ciufo S, Haddad D, Mcveigh R (2016). Reference sequence (RefSeq) database at NCBI: Current status, taxonomic expansion, and functional annotation. Nucleic Acids Res.

[64] Luo E, Eppley JM, Romano AE, Mende DR, DeLong EF (2020). Double-stranded DNA virioplankton dynamics and reproductive strategies in the oligotrophic open ocean water column. ISME J.

[65] Mizuno CM, Rodriguez-Valera F, Kimes NE, Ghai R (2013). Expanding the marine virosphere using metagenomics. PLoS Genet.

[66] Mizuno CM, Ghai R, Saghaï A, López-García P, Rodriguez-Valera F (2016). Genomes of abundant and widespread viruses from the deep ocean. MBio..

[67] Roux S, Brum JR, Dutilh BE, Sunagawa S, Duhaime MB, Loy A (2016). Ecogenomics and biogeochemical impacts of uncultivated globally abundant ocean viruses. Nature..

[68] Paez-Espino D, Eloe-Fadrosh EA, Pavlopoulos GA, Thomas AD, Huntemann M, Mikhailova N (2016). Uncovering Earth’s virome. Nature..

[69] López-Pérez M, Haro-Moreno JM, Gonzalez-Serrano R, Parras-Moltó M, Rodriguez-Valera F (2017). Genome diversity of marine phages recovered from Mediterranean metagenomes: Size matters. PLoS Genet.

[70] Coutinho FH, Silveira CB, Gregoracci GB, Thompson CC, Edwards RA, Brussaard CPD (2017). Marine viruses discovered via metagenomics shed light on viral strategies throughout the oceans. Nat Commun.

[71] Gregory AC, Zayed AA, Sunagawa S, Wincker P, Sullivan MB, Ferland J (2019). Marine DNA viral macro- and microdiversity from pole to pole. Cell..

[72] Luo E, Aylward FO, Mende DR, Delong EF (2017). Bacteriophage distributions and temporal variability in the ocean’s interior. MBio..

[73] Eren AM, Esen ÖC, Quince C, Vineis JH, Morrison HG, Sogin ML (2015). Anvi’o: An advanced analysis and visualization platform for ‘omics data. PeerJ..

[74] Langfelder P, Horvath S. WGCNA: An R package for weighted correlation network analysis. BMC Bioinformatics. 2008;9:559. 10.1186/1471-2105-9-559 PMC2631488 19114008

[75] R Core Team. R: A Language and Environment for Statistical Computing [Internet]. Vienna, Austria; 2019. Available from: https://www.r-project.org/

[76] Lauro FM, Chastain RA, Blankenship LE, Yayanos AA, Bartlett DH (2007). The unique 16S rRNA genes of piezophiles reflect both phylogeny and adaptation. Appl Environ Microbiol.

[77] DeLong EF, Franks DG, Yayanos AA (1997). Evolutionary relationships of cultivated psychrophilic and barophilic deep-sea bacteria. Appl Environ Microbiol.

[78] Berg KA, Lyra C, Sivonen K, Paulin L, Suomalainen S, Tuomi P (2009). High diversity of cultivable heterotrophic bacteria in association with cyanobacterial water blooms. ISME J.

[79] Rii YM, Karl DM, Church MJ (2016). Temporal and vertical variability in picophytoplankton primary productivity in the North Pacific Subtropical Gyre. Mar Ecol Prog Ser.

[80] Martin JH, Knauer GA, Karl DM, Broenkow WW (1987). VERTEX: Carbon cycling in the northeast Pacific. Deep-Sea Res.

[81] Karl MD, Knauer AG (1984). Detritus-microbe interactions in the marine pelagic environment: Selected results from the vertex experiment. Bull Mar Sci.

[82] Scanlan DJ, Ostrowski M, Mazard S, Dufresne A, Garczarek L, Hess WR (2009). Ecological genomics of marine picocyanobacteria. Microbiol Mol Biol Rev.

[83] McDonnell AMP, Boyd PW, Buesseler KO (2015). Effects of sinking velocities and microbial respiration rates on the attenuation of particulate carbon fluxes through the mesopelagic zone. Glob Biogeochem Cycles.

[84] Qiu B, Koh DA, Lumpkin C, Flament P (1997). Existence and formation mechanism of the North Hawaiian Ridge Current. J Phys Oceanogr.

[85] Turner JT (2015). Zooplankton fecal pellets, marine snow, phytodetritus and the ocean’s biological pump. Prog Oceanogr.

