# Supplementary material for: Diversity and origins of bacterial and archaeal viruses on sinking particles reaching the abyssal ocean
Source: ISME J. 2022 Mar 2;16(6):1627–35. doi: 10.1038/s41396-022-01202-1 (PMC9122931; doi:10.1038/s41396-022-01202-1)
Supplement: Supplementary file 1 — Supplementary figures+legends, supplementary table legends [file 41396_2022_1202_MOESM1_ESM.docx]

**Supplementary figures**

**Figure S1. Bioinformatic workflow** from metagenomic reads to DTV database and analyses.

**Figure S2. Particulate carbon export flux and primary productivity** (12-hour light incubations) through the 3-year sampling period. Solid line indicates the 30-year mean, while the dashed line indicates 150% of the 30-year mean, as previously reported (1,2). Grey shading indicates carbon-based summer export pulse samples. Particulate carbon export data is courtesy of Eric Grabowski and David Karl. Productivity data from 12-hour light incubations was retrieved from http://hahana.soest.hawaii.edu/hot/hot-dogs/

**Figure S3. Spatiotemporal coverage abundance profiles of 21 viruses captured in sinking particles that we postulate originated from the upper 500 m.** Each node on the top dendrogram and its associated column represents the coverages of one viral population. Each row represents an individual sample from the ALOHA2.0 dataset collected from the upper 500m at Station ALOHA (3). All samples are by depth, and within depth, ordered by time. The height of black bars represents log IQR coverage for each population, normalized to the maximum in that sample. The bottom blue bar highlights viruses that correlate with particulate carbon export flux (group color consistent with Fig. 5).

**
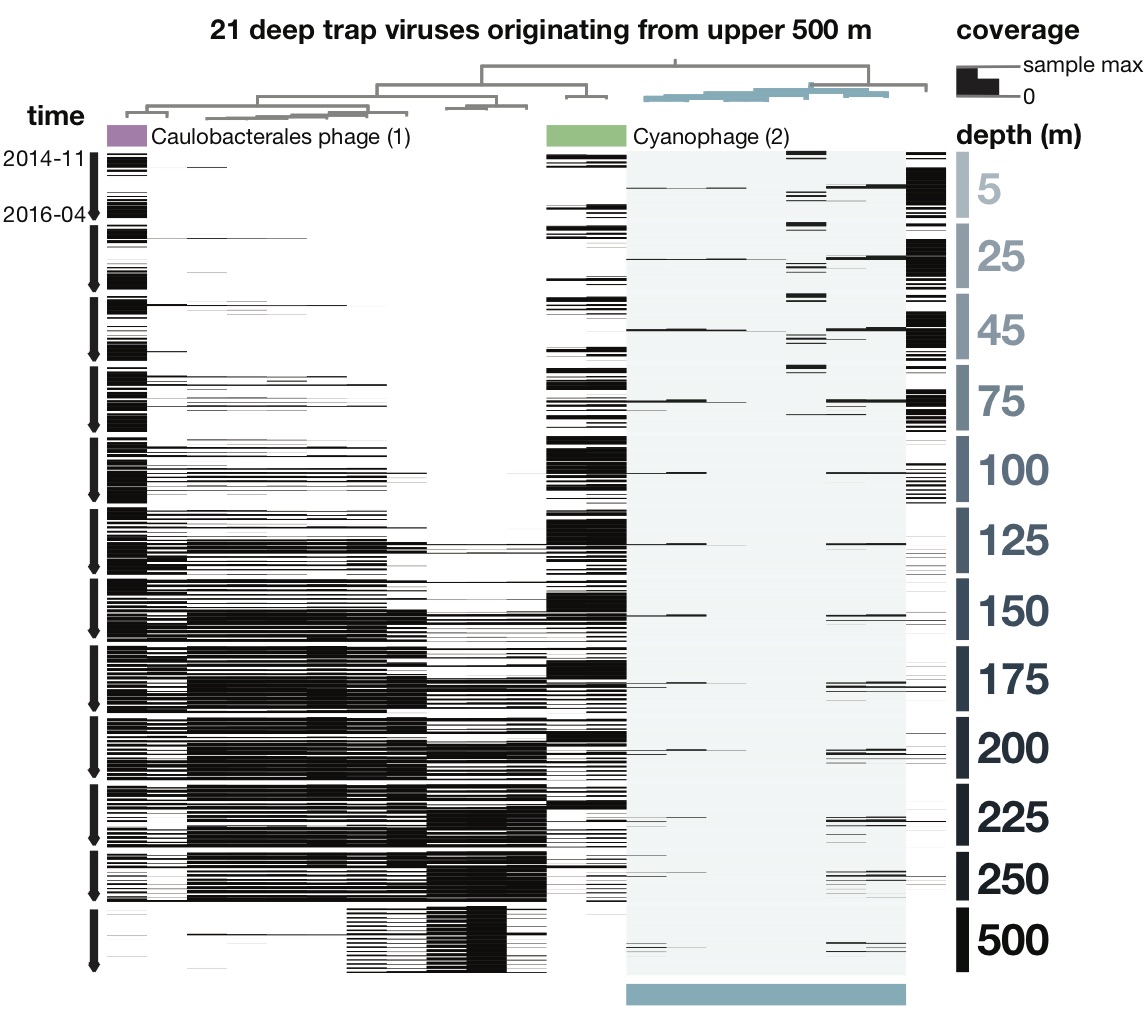
**

**Figure S4. Examples of different virus-host abundance patterns observed in DTVs**: a. Phage-host abundance co-variance and b. c. Decoupled phage-host abundances. The left panels display abundances of viruses (closed circles) and hosts (open circles) through time. Normalized coverage was calculated using IQR coverage for each viral population genome and MAG bin, normalized to the smallest sequenced sample. IQR coverages amongst multiple scaffolds within each MAG population bin were average-weighted by scaffold length. The right panels display viruses and their position in MAG scaffolds, with viral structural proteins colored in orange, integrases in yellow, and protein alignments in blue. Below, coverage along MAG scaffold is shown for two samples corresponding to time-points labeled on the left panel (green and lavender triangles). Black rectangles highlight viral regions in MAG scaffolds that display coverage discrepancies relative to non-viral regions.


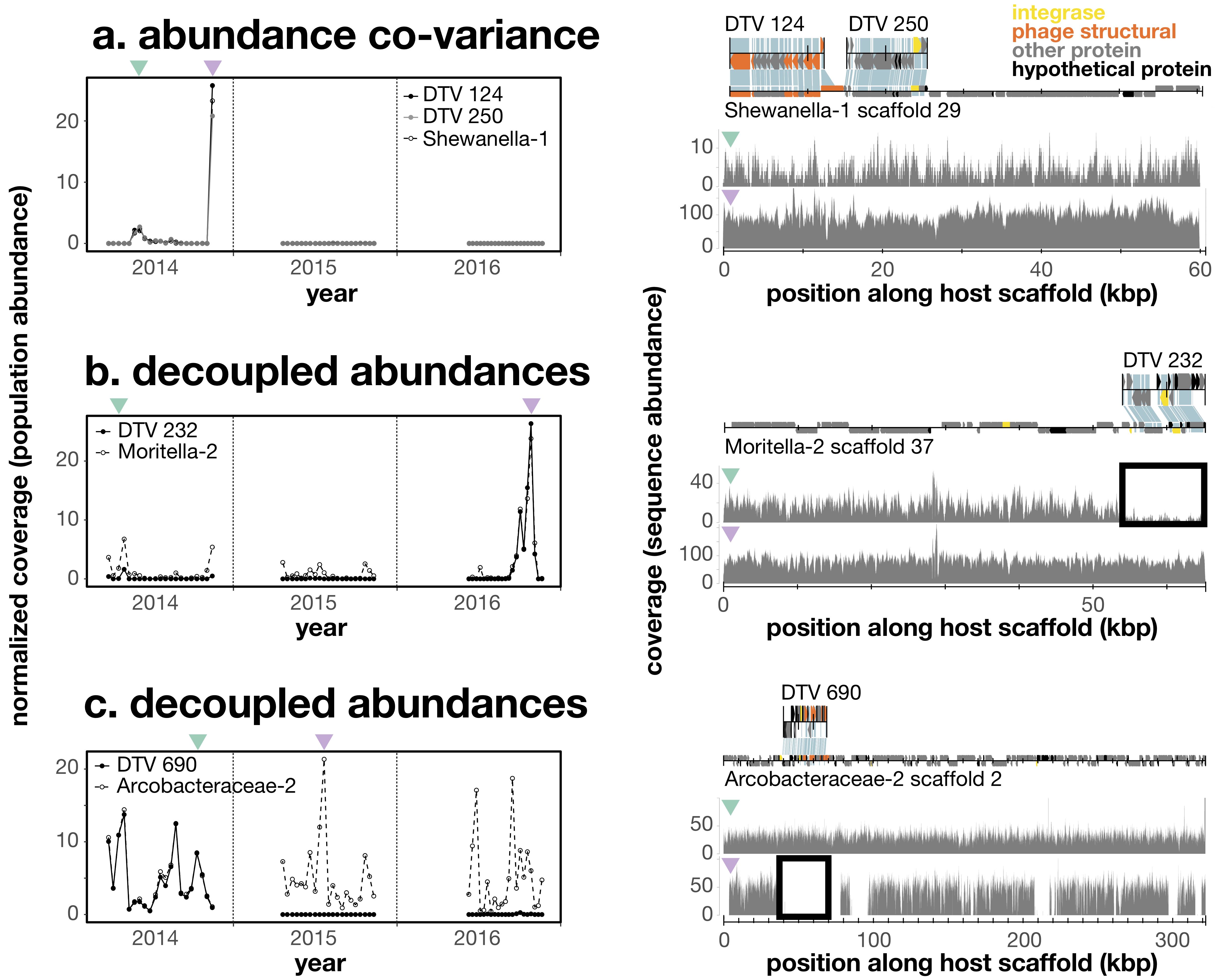


**Supplementary tables**

**Table S1. Sequencing, assembly, and viral contig identification** for 63 metagenomic samples (1). Columns represent: sample name, number of quality-controlled reads, number of contigs, number of contigs >1kb for input into viral identification programs, number of viral contigs identified by VIRSorter, number of viral contigs identified by both VIRSorter and VIBRANT, number of viral contigs identified by VIBRANT, number and proportion of contigs identified by VIRSorter but not by VIBRANT, number and proportion of contigs identified by VIBRANT but not by VIRSorter.

**Table S2. Information on 857 DTVs.** Columns represent: name, length, GC content, genomic completion, chimeric signature, temperate, host prediction by alignments to MAGs, GTDB, RefSeq, and ALOHA2.0 (3), novelty with respect to previously sequenced viral metagenomes, WGCNA group that correlate with particulate carbon flux (Fig. 3, bottom panel), number of samples present out of 63 total deep trap samples, presence in 2015 150 m particle samples, presence in 2017 150 m particle samples, presence in 5 – 500 m planktonic samples, and presumptive depth of origin (3).

**Table S3. Alignments between DTVs and MAGs**. Columns are in BlastTab format and represent: DTV, DTV length, MAG scaffold, MAG scaffold length, percent nucleic acid identity, length of alignment, mismatches, gap opens, start of alignment on DTV, end of alignment on DTV, start of alignment on MAG scaffold, end of alignment on MAG scaffold, e-value, and bit score.

**Table S4.** **Alignments between 21 DTV and the ALOHA 2.0 viral database** of viruses recovered from upper 500 m samples in the same environment (3). Columns are in BlastTab format and represent: DTV, DTV length, ALOHA2.0 virus, ALOHA2.0 virus length, taxonomy based on >=60% AAI across >=50% of proteins, percent nucleic acid identity, length of alignment, mismatches, gap opens, start of alignment on DTV, end of alignment on DTV, start of alignment on ALOHA2.0 virus, end of alignment on ALOHA2.0 virus, e-value, and bit score.

**Table S5.** **Novel PFAM protein domains (bit score >30) recovered from the DTV database** that were not found in previously reported datasets (3–5).

**Table S6. Relative abundances of 857 DTVs** approximated by IQR coverage normalized to the smallest library size (normalized coverage).

**Table S7.** **Relative abundances of 129 cellular MAGs** approximated by IQR coverage weighted by scaffold length for each MAG and normalized to the smallest library size (normalized coverage).

**References**

1. Poff KE, Leu AO, Eppley JM, Karl DM, DeLong EF. Microbial dynamics of elevated carbon flux in the open ocean’s abyss. Proc Natl Acad Sci U S A. 2021;118(4):1–11.

2. Karl DM, Church MJ, Dore JE, Letelier RM, Mahaffey C. Predictable and efficient carbon sequestration in the North Pacific Ocean supported by symbiotic nitrogen fixation. Proc Natl Acad Sci USA. 2012;109(6):1842–9.

3. Luo E, Eppley JM, Romano AE, Mende DR, DeLong EF. Double-stranded DNA virioplankton dynamics and reproductive strategies in the oligotrophic open ocean water column. ISME J. 2020;14(5):1304–15.

4. Roux S, Brum JR, Dutilh BE, Sunagawa S, Duhaime MB, Loy A, et al. Ecogenomics and biogeochemical impacts of uncultivated globally abundant ocean viruses. Nature. 2016;537:689–93.

5. Luo E, Aylward FO, Mende DR, Delong EF. Bacteriophage distributions and temporal variability in the ocean’s interior. MBio. 2017;8(6):e01903-17.
